# Metabolomic-Based Study of the Leafy Gall, the Ecological Niche of the Phytopathogen *Rhodococcus Fascians*, as a Potential Source of Bioactive Compounds

**DOI:** 10.3390/ijms140612533

**Published:** 2013-06-14

**Authors:** Aminata P. Nacoulma, Olivier M. Vandeputte, Manuella De Lorenzi, Mondher El Jaziri, Pierre Duez

**Affiliations:** 1 Laboratory of Toxicology, Faculty of Pharmacy, Université Libre de Bruxelles, CP 205/1, Boulevard du Triomphe, Brussels B-1050, Belgium; E-Mail: manuela.de.lorenzi@ulb.ac.be; 2 Laboratory of Plant Biotechnology, Faculty of Sciences, Université Libre de Bruxelles, 12 rue des Professeurs Jeener et Brachet, Gosselies B-6041, Belgium; E-Mails: olivier.vandeputte@ulb.ac.be (O.M.V.); jaziri@ulb.ac.be (M.E.J.); 3 Laboratory of Pharmacognosy, Bromatology and Human Nutrition, Faculty of Pharmacy, Université Libre de Bruxelles, CP 205/9, Boulevard du Triomphe, Brussels B-1050, Belgium; E-Mail: pduez@ulb.ac.be

**Keywords:** metabolomics, tobacco, multivariate data analysis, diterpenoids, leafy gall, *Rhodococcus fascians*

## Abstract

Leafy gall is a plant hyperplasia induced upon *Rhodococcus fascians* infection. Previously, by genomic and transcriptomic analysis, it has been reported that, at the early stage of symptom development, both primary and secondary metabolisms are modified. The present study is based on the hypothesis that fully developed leafy gall, could represent a potential source of new bioactive compounds. Therefore, non-targeted metabolomic analysis of aqueous and chloroform extracts of leafy gall and non-infected tobacco was carried out by ^1^H-NMR coupled to principal component analysis (PCA) and orthogonal projections to latent structures-discriminant analysis (OPLS-DA). Polar metabolite profiling reflects modifications mainly in the primary metabolites and in some polyphenolics. In contrast, main modifications occurring in non-polar metabolites concern secondary metabolites, and gas chromatography and mass spectrometry (GC-MS) evidenced alterations in diterpenoids family. Analysis of crude extracts of leafy galls and non-infected tobacco leaves exhibited a distinct antiproliferative activity against all four tested human cancer cell lines. A bio-guided fractionation of chloroformic crude extract yield to semi-purified fractions, which inhibited proliferation of glioblastoma U373 cells with IC_50_ between 14.0 and 2.4 μg/mL. Discussion is focused on the consequence of these metabolic changes, with respect to plant defense mechanisms following infection. Considering the promising role of diterpenoid family as bioactive compounds, leafy gall may rather be a propitious source for drug discovery.

## 1. Introduction

Plant galls are tumors triggered by external aggressions, especially microorganisms or insects [1]. Although galls display many phenotypes, they provide niches to protect and ensure the survival of the pathogen [2,3]. In the case of bacteria, the interaction involves a complex chemical exchange contributed by and specific to both the host and the pathogen [4,5]. Galls are of great medicinal value and have widely been used in traditional medicines. Therefore, prospecting for chemicals that are specifically induced in plant galls is a promising alternative for drug discovery [6–8]. For instance, *Rhodococcus fascians*, a phytopathogenic soil Gram-positive actinomycete has been shown to produce phytohormones [5,9,10], triggering plant cell divisions and signal transduction. This leads to cell reprogramming [11,12], resulting in the development of the so-called leafy galls on a wide range of host plants [8]. At the early stage of leafy gall development, both primary and secondary metabolisms are modified to contribute to symptoms development [12]. However, the metabolic changes characterizing the fully developed leafy gall, which is the ecological niche of the *R. fascians*, remain largely unexplored, except for the identification of 7-methyl-esculin, a phenolic compound accumulated in tobacco symptomatic tissues, but not in non-infected tissues [13].

Actinomycetes are a rich and highly exploited source of antibiotics and other bioactive secondary metabolites of medical or industrial interest [14,15]. Similarly, medicinal plants have been used since prehistory to treat human diseases or to improve human health and today plant drug discovery remains extremely important for the pharmaceutical industry [16,17]. Recently, non-pathogenic actinomycetes, living inside plant tissues as endophytes, are being screened for the production of bioactive compounds [18,19]. However, plant tissue hyperplasia induced upon interaction with pathogenic bacteria are, to our knowledge, an unexplored yet unique resource for new compounds with promising potential for drug development. With the availability of non-targeted methods, biological samples can be characterized by innovative approaches; metabolomic methods, based on gas or liquid chromatography and mass spectrometry (GC-MS and LC-MS), or on nuclear magnetic resonance (NMR), generate comprehensive metabolic fingerprints of metabolites being modified during a biological process or occurring in symptomatic tissues [20].

Here, the monitoring of the metabolic status was investigated by NMR and antiproliferative activity by MTT assay, comparing polar and non-polar extracts between well-developed leafy galls and non-infected tobacco tissues. The NMR data analyses were performed with an unsupervised clustering method (principal component analysis, PCA) and, when appropriate, by a supervised method (orthogonal projections to latent structures-discriminant analysis, OPLS-DA).

## 2. Results and Discussion

### 2.1. The Morphological Features of Plant Material

**Figure 1:**
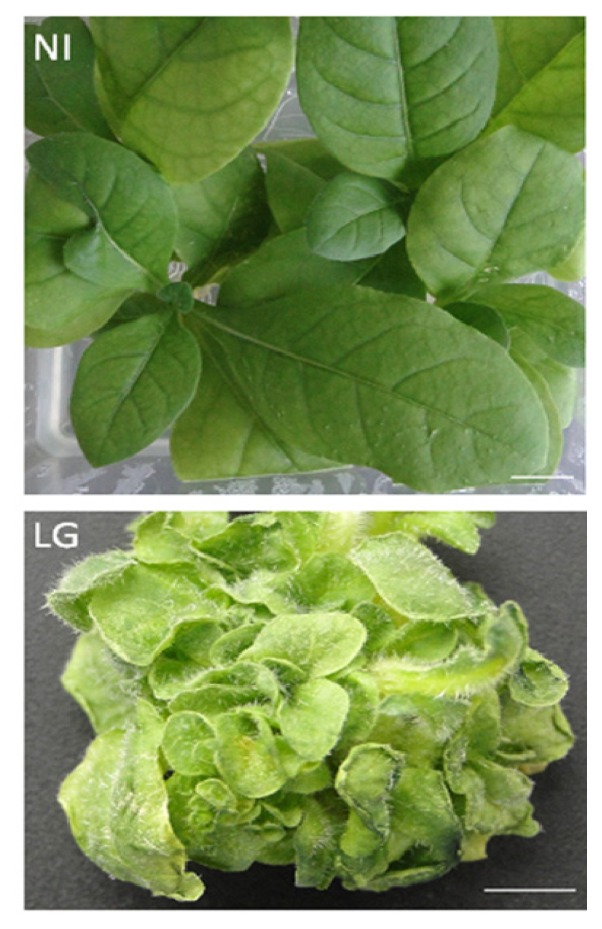
The morphological features of leafy gall (LG) formed in *Rhodococcus fascians*-infected tobacco plant (eight weeks post infection) and non-infected (NI) tobacco plant grown for 12 weeks. The *in vitro* tobacco plants were infected four weeks post germination. Bar = 1 cm.

Leafy galls (LG) were induced in four-week-old *in vitro* growing tobacco plants following infection with the virulent *R. fascians* strain D188. Figure 1 illustrates the phenotype of the non-infected (NI) tobacco plants 11 weeks post germination and a typical eight-week-old LG, a morphological structure with multiple dormant buds and malformed leaves characterized by increased trichomes formations.

### 2.2. Characteristics of ^1^H NMR Spectra of Polar and Non-Polar Tobacco Extracts

**Figure 2:**
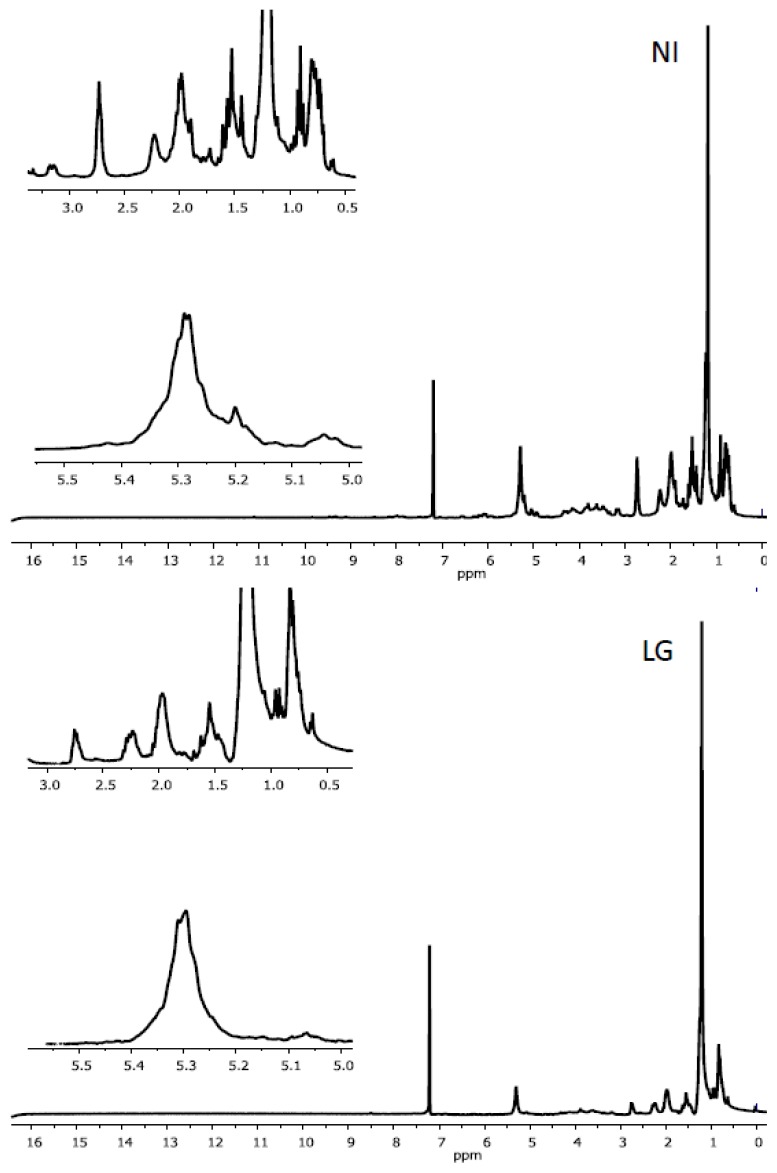
The typical ^1^H-NMR spectra (300.13 MHz) of non-polar tobacco metabolites from NI and LG tissues. Zoom-ups of the aliphatic (0.5–3.0 ppm) and olefinic (5.0–5.5 ppm) regions are included.

**Figure 3:**
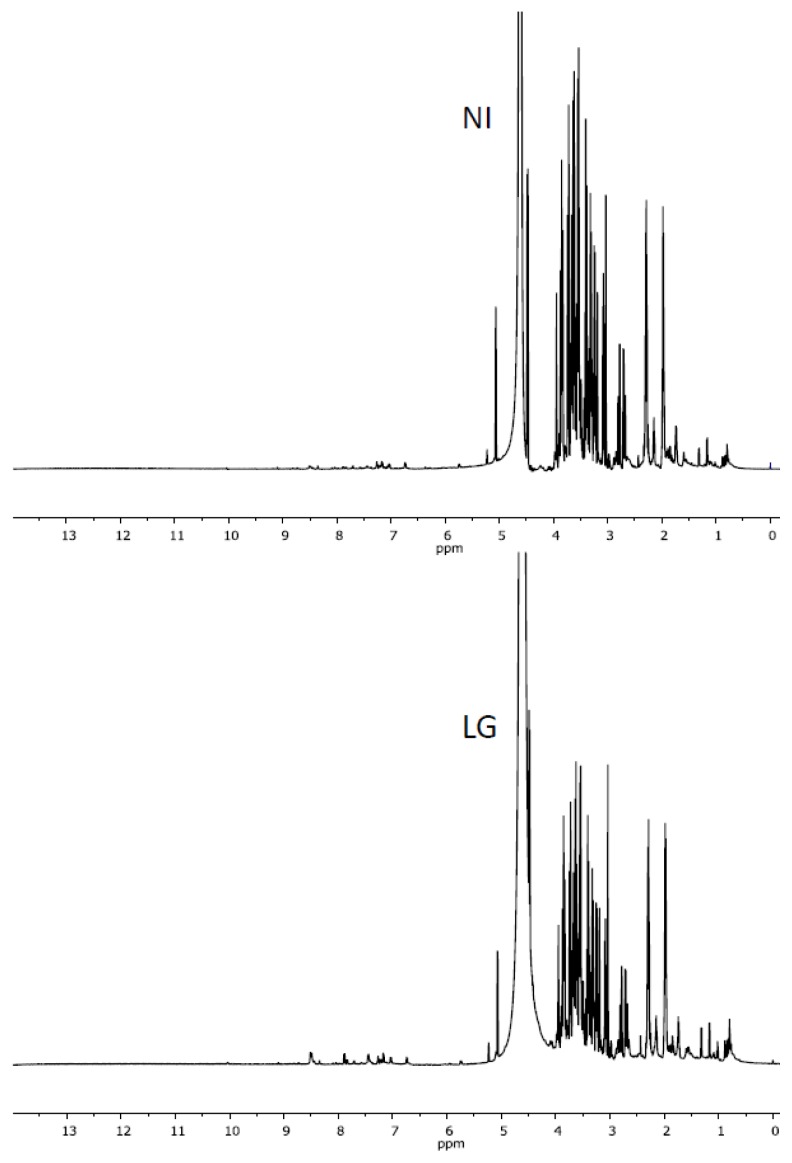
The typical ^1^H-NMR spectra (300.13 MHz) of polar tobacco metabolites from NI and LG infected tissues.

As shown in Figure 2, visual inspection of the ^1^H-NMR spectra of non-polar metabolites (chloroformic extracts) revealed roughly similar chemical shift patterns in the aromatic region (δ 7–8 ppm) but striking differences in both the olefinic (δ 4.9–6.5 ppm) and aliphatic (δ 0–3 ppm) regions. According to Qin [21], the methyl signals located at δ 0.7–1.3 ppm might originate from steroids or terpenoids and, at δ 1.2–1.4 ppm, from fatty components. Thus, the significant signals observed at δ 1.5–3.0 ppm and at δ 5.0–5.5 ppm might be assigned to methylene group from sesquiterpenes or diterpenes and to olefinic signals from fatty components, steroids, or terpenoids, respectively. These data suggest an alteration in biosynthetic pathways leading to the accumulation of terpenoids and/or fatty compounds to the detriment of the metabolites diversity in infected tobacco cells. In contrast, a visual observation of the ^1^H-NMR spectra of polar metabolites (aqueous extracts) revealed no obvious differences in chemical shifts between *R. fascians* infected (LG) and non-infected (NI) tobacco plants (Figure 3).

### 2.3. Statistical Analysis of the Non-Polar Metabolites

**Figure 4:**
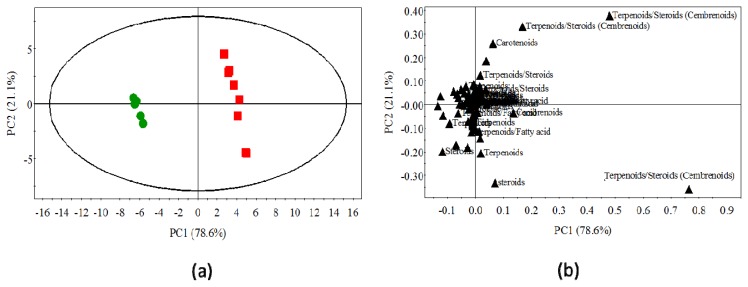
Principal components analysis (PCA) of ^1^H-NMR spectra for chloroformic extracts of both LG and NI tobacco tissues; (**a**) The first two components explain 99.7% of the variation (PC1: 78.6% and PC2: 21.1%) with a clear discrimination between LG and NI extracts along PC1; (**b**) Loading plot of the PC1 component shown in **a**. The discrimination between infected and non-infected tobacco plant extracts mainly takes place in the aliphatic region, at δ 0.7–3.0 ppm, and in the olefinic region, at δ 5.0–5.5 ppm. These spectral regions correspond to the signals characteristic of terpenoids and steroids [21].

**Figure 5:**
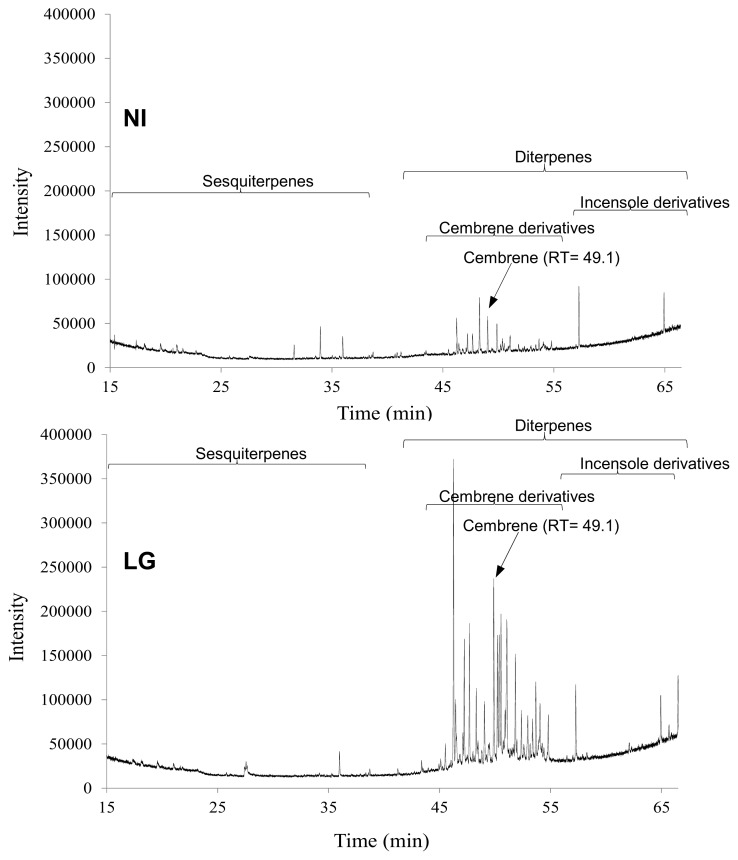
The typical GC-MS chromatograms of non-polar tobacco metabolites from NI and LG tobacco tissues. The arrow indicates the gas chromatography and mass spectrometry (GC-MS) peak corresponding to commercially available cembrene. See Material and Methods for chromatographic conditions.

As shown in the score plot (Figure 4a), the first two principal components explain 99.7% of the variability in the dataset (PC1: 78.6% and PC2: 21.1%) and clearly identify two different clusters of samples. These PCA scores were tested by a one-way-ANOVA (Table 1), indicating significant differences between *R. fascians*-infected and non-infected tobacco metabolites profiles (*p* < 0.001). According to the mapping metabolite approach described by Graham [22], the PC1 component allows an obvious discrimination between extracts of *R. fascians*-infected and non-infected tobacco plants. Examination of the loadings plot (Figure 4b) shows that the variance along the first principal component is mainly driven by modifications in the aliphatic (δ 0.7–3 ppm) and the olefinic (δ 5.0–5.5 ppm) regions. These PC1 discriminating signals confirm that low-polarity secondary metabolites, possibly steroids or terpenoids, could account for some major changes occurring in tobacco plants following *R. fascians* infection. To further support this assumption, qualitative GC-MS analysis of chloroformic extracts from both LG and non-infected plants was investigated. Figure 5 shows major differences in the GC chromatograms, particularly in compounds eluted from 45 to 60 min., which, according to Hamm [23], correspond to tobacco diterpenoids, such as cembrenoids, as illustrated by the retention time of commercially available cembrene (at 49.1 min) which are found to be accumulated more than two times in LG as compared to NI extracts. It is noteworthy to mention that cembrenoids and closely related metabolites are found to accumulate in tobacco trichomes [24], a characteristic anatomical feature of leafy galls (Figure 1). It is therefore tempting to postulate that, diterpene biosynthetic pathway and particularly cembrenoids family is stimulated in infected tobacco tissue.

The non-polar metabolites fingerprinting reveals that the *R. fascians* infection induces the expression of secondary metabolites with aliphatic signals consistent with the induction and accumulation of terpenic compounds. The qualitative GC analyses of the LG and NI tobacco extracts, according to a procedure described in the literature for the profiling of terpenoids [23], indicates that induced compounds could belong to the tobacco diterpene family. This confirms a previous observation by transcriptomic and BiNGO (Biological Network Gene Ontology) analysis of up-regulated genes in *R. fascians* infected-*Arabidopsis* that revealed an up-regulation of the plant host diterpenoid pathway [12]. To date, the role of terpenoid compounds in the *R. fascians*-plant interactions remains unknown, although some tobacco-pathogen interactions are known for leading to alterations in the terpenes pathway. The induction of the monoterpene E-(*b*)-ocimene and the sesquiterpenes caryophyllene, β-elemene and α-farnesene, have been shown during the interaction of *Nicotiana tabacum* with *Golovinomyces cichoracearum* [25] and *Pseudomonas syringae* [26], respectively. Seo [27] identified a diterpene, the (11E,13E)-labda-11,13-diene-8α,15-diol, as an endogenous signal for the activation of tobacco defense responses to wounding and to infection with the tobacco mosaic virus; many terpenoids are known for their function as phytoalexins, contributing to plant defenses against microbes [28].

### 2.4. Statistical Analysis of Polar Metabolites

**Figure 6:**
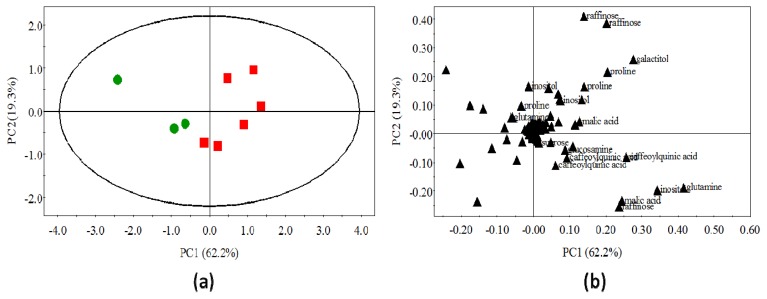
The principal components analysis of ^1^H-NMR spectra for aqueous extracts of both NI and LG tobacco tissues; (**a**) The first two components explain 81.5% of the variation (PC1: 62.2% and PC2: 19.3%) with a small discrimination between NI and LG extracts along PC1; (**b**) Loading plot of the PC1 component shown in (a). The discrimination between infected and non-infected tobacco plant extracts mainly takes place at δ 2–5.5 ppm.

As shown in Figure 6a, the first two principal components explain 81.5% of the variability in the dataset (PC1: 62.2% and PC2: 19.3%) and could not identify samples clusters. PCA scores were tested by a one-way-ANOVA (Table 1), indicating non-significant difference between *R. fascians*-infected and non-infected tobacco metabolites profiles (*p* > 0.05). Examination of the PCA loadings plot (Figure 6b) shows that the variance along the first principal component is mainly driven by modifications in the region δ 2.0–5.5 ppm. To avoid any biased interpretation of group discrimination based on PCA analysis, the dataset was reanalyzed using OPLS-DA, a supervised method based on an *a priori* knowledge of distinct samples.

**Figure 7:**
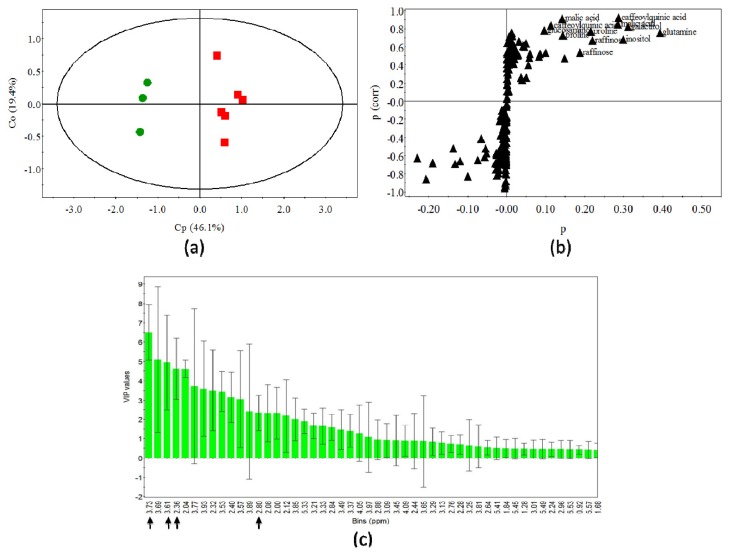
The orthogonal projections to latent structures discriminating analysis (OPLS-DA) of ^1^H-NMR spectra for aqueous extracts of both NI and *Rhodococcus fascians*-infected LG tobacco tissues; (**a**) The predictive component (Cp, *X*-axis) explains 46.1% and the first orthogonal component (Co1, *Y*-axis) 19.4% of the dataset variation; (**b**) The *X* axis (p) describes the loadings of each variable and *Y*-axis (p(corr)) represents the reliability of each variable in the dataset. Cut-off values for the covariance and the correlation were assigned to *p* > 0.05 and *p*(corr) > 0.5, respectively (S-plot). Metabolites in the upper-right and lower-left quadrants are potential biomarkers, and best discriminate the LG and NI samples with smaller risk for illegitimate correlations (*p* > 0.05); the induced compounds corresponding to discriminant loadings are inferred according to the literature [21,30]. These include sugars (raffinose, galactitol), aminoacids (proline, glutamine), organic acids (malic acid), and phenolics (caffeoylquinic acid); (**c**) OPLS-DA variable importance in the projection-plot with confidence intervals of buckets contributing to discriminating LG and NI tobacco plants polar extracts; the arrows indicate the ^1^H chemical shifts (ppm) of significant polar metabolites (Table 3).

As shown in Table 2, the comparisons between samples in models gave better predictions in OPLS-DA compared to PCA analysis (predictability of the total model: Q^2^ OPLS-DA > Q^2^ PCA; 0.93 and 0.65, respectively). OPLS-DA facilitates the identification of the different sources of variation that contribute to the differences between the extracts, by separating inter-group variation (*i.e.*, variation that is predictive of differences between LG and NI tissues) from intra-group variation (*i.e.*, variation that is unrelated to group separation). As shown in Figure 7a, a part of the total variance (predictive component, Cp 46.1%) is predictive, being specifically related to the differences induced by *R. fascians* infection. In contrast, the first orthogonal component (Co) describing 19.4% of the total variation in the dataset is responsible of the high variability within the dataset. The discriminating signals were interpreted through a loading S-plot (Figure 7b) that allows distinguishing of both the covariance (contribution of the effect) and the correlation (reliability of the predicted variables) for the identification of the major contributors in samples discrimination. In this OPLS model, increased metabolites correspond to those that show a high (i) correlation with the predictive scores, (*p*(corr) > 0.5) and (ii) co-variation with the predictive scores (*p* > 0.05), with respect to the component scores model. At these cut-off levels, only the metabolites identified by the OPLS-DA model were considered as significant contributors [29]. In OPLS-based regression, VIP values (variable importance in the projections) are directly proportional to the influence of variables on the separation on score plots. Variables with higher VIP values are more important for the samples discrimination. In the OPLS-DA analysis of LG and NI tobacco plants extracts, VIP values for the main metabolites, responsible for separation on the score plot, are represented in Figure 7c and Table 3. The high VIP scores identified for proline, inositol, malic acid, and caffeoylquinic acid, legitimate their occurrence in the well-developed LG, as compared to NI tissues.

The polar metabolites fingerprinting shows that metabolites discriminating LG from NI tobacco plants are likely primary metabolites, such as sugars (raffinose, galactitol), amino acids (proline, glutamine), and some organic and phenolic acids (malic acid, caffeoylquinic acid derivatives), suggesting a role of these compounds in the established ecological niche of *R. fascians.* Synthesis of primary metabolites seems to be a quite general mechanism, observed during plant-pathogen interactions [31,32], and the response of *N. tabacum* is probably not specific to this interaction. The increase in sugar contents in LG may reflect the energy costs linked to the activation of the plant defenses [33] or a redirection of the host plant metabolism, presumably to the *R. fascians* own advantage [12]. The increase in some amino acids, such as proline and glutamine, may contribute to the pathogenicity of *R. fascians* by providing appropriate conditions for the expression of virulence factors [12]. Interestingly though, the third main discriminatory class of molecules emerging from our study are organic acids and phenols (malic acid, caffeoylquinic acid, and kaempferol), pointing to a possible defense reaction occurring in LG; this is consistent with the induction of defense-related genes observed in developed LG of *Atropa belladonna*, tobacco and *Arabidopsis* [12,34,35]. Indeed, the phenylpropanoid pathway is known to be induced in response to a pathogen attack [36], and several phenolics are known for their potential anti-bacterial activities. For instance, caffeoylquinic acid derivatives were recently identified as antimicrobials and inhibitors of efflux pump of Gram-positive pathogenic bacteria [37].

### 2.5. Leafy Gall (LG) Tissues Contain Potent Compounds That Affect the Proliferation of Different Human Cancer Cell Lines

The occurrence of antiproliferative activity in LGs and NI was assessed in crude water, hexane, methanol, and chloroform extracts by determining the concentration of extract required to obtain a 50% inhibition (IC_50_) of the proliferation of four different cancer cell lines, U373, A549, MCF-7, and PC-3 using MTT assays. The hexane, methanol, and water extracts of the LGs and the NI extracts did not affect any of the cell lines (Table 4). In contrast, the chloroformic extract of the LGs, but not of NIs, exhibited a distinct antiproliferative activity against all four tested cell lines, albeit to different extents.

The proliferation of line U373 was affected most strongly (IC_50_ = 58.9 ± 2.0 μg/mL), followed by that of line A549 (IC_50_ = 69.8 ± 0.9 μg/mL), whereas lines MCF-7 and PC-3 had a lower but comparable sensitivity for the crude extract (IC_50_ = 84.5 ± 4.3 μg/mL and 85.6 ± 1.0 μg/mL, respectively).

**Figure 8:**
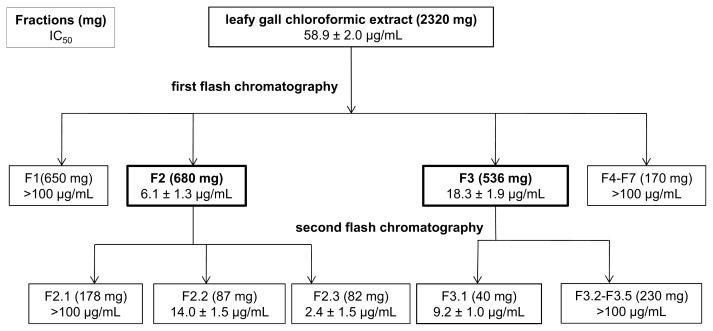
The crude chloroformic extract of tobacco leafy galls contain compounds with antiproliferative activity against the glioblastoma U373 cell line. Schematic overview of the bio-guided fractionation of LG crude extract using the MTT colorimetric cell growth inhibition bioassay. The efficiency of each considered fraction is expressed as the IC_50_ value; fractions in bold were analyzed further. The data are presented as the mean ± SD (*n* = 3).

Because the glioblastoma cell line U373 exhibited the strongest sensitivity toward the LG extract, this line was chosen for all further experiments. As a first step in the chemical characterization of the bioactive compound(s) present in the LGs, the crude chloroformic extract was fractionated and the IC_50_ was determined for each fraction. As shown in Figure 8, a first chromatographic fractionation yielded two out of seven fractions with IC_50_ values below the value obtained for the crude extract (F2 and F3). These fractions were subjected to a second chromatography fractionation allowing the isolation of three active sub-fractions (F2.2, F2.3 from F2, and F3.1 from F3). These preliminary results on the inhibition of human tumor cell growth indicate that fully developed leafy gall, could represent a potential source of new bioactive compounds.

Because none of the tested extracts from NI exhibited antiproliferative activity, the biological activity observed in the chloroformic extract of LGs has to be associated with the reprogramming of the development and metabolism of the host, essentially the diterpenoids family (Figure 5) since such compounds have already been reported to inhibit cell proliferation [38].

## 3. Experimental Section

### 3.1. Solvents and Chemicals

Analytical grade chloroform was purchased from Chemlab (Redu, Belgium). CDCl_3_ (99.96%) and D_2_O (99.0%) with buffering agent (pH 6.5) were purchased from Sigma Aldrich (St. Louis, MO, USA).

### 3.2. Plant Material and Bacterial Infection

Tobacco plants (*Nicotiana tabacum* L. cv. Petit Havana) were aseptically grown from sterile seeds (obtained at the Jean Massart Botanical Garden from the Université Libre de Bruxelles) on solid half-strength MS medium (Duchefa), supplemented with 3% sucrose, in a growth chamber at 23 °C in a 16-h light photoperiod (70 μmol photonsm^−2^s^−1^). Four week-old plants were injured and infected at the apical bud with 10 μL of *R. fascians* virulent cultures strain D188 according to Rajaonson *et al* [39]. The leafy galls (LG) that formed were harvested eight weeks after infection. Non-infected (NI) tobacco plants were harvested after twelve weeks of growth (at the same developmental stage as the infected plants).

### 3.3. Established Cell Lines

The human U373 (ATCC code HTB-17) glioblastoma, A549 (DSMZ code ACC107) non-small cells lung carcinoma, MCF-7 (DSMZ code ACC115) breast cancer and PC-3 (DSMZ code ACC465) prostate cancer cell lines were obtained from the American Type Culture Collection (ATCC; Manassas, VA, USA) and maintained in our laboratory as described previously [40].

### 3.4. Extraction of Plant Material

#### 3.4.1. For Nuclear Magnetic Resonance (NMR) Analysis

Fifteen (for chloroform extraction) and nine (for aqueous extraction) independent samples, of infected and non-infected plants were randomly constituted by mixing material harvested from plants. Each sample was ground in liquid nitrogen and freeze-dried. Three hundred milligrams aliquots of each sample were transferred into centrifuge tubes. Five milliliters of chloroform or water were added to the tube followed by rapid vortexing and sonication for 30 min. The material was then centrifuged at 3000 rpm for 15 min. The extraction was repeated 2 times and the combined extracts were transferred into a 25-mL round bottom flask and dried with a rotary vacuum evaporator (40 °C for chloroform and 70 °C for water). The residue was dissolved in 1 mL of CDCl_3_ or D_2_O, respectively, and subjected to ^1^H-NMR analysis.

#### 3.4.2. Chromatgraphic Fractionnaltion of LG and Non-Infected (NI) Extracts for Antiproliferative Activity Measurment

Each of LG or NI material was grinded in liquid nitrogen, freeze-dried, and extracted 3 times with 500 mL of water, hexane, methanol, or chloroform (analytical grade Chemlab, Belgium). Subsequently, the chloroformic dry crude extract (2320 mg), obtained from 1.03 kg of LG material, was subjected to two successive automated flash chromatography systems (CombiFlash^®^ Rf 200 psi from Teledyne isco^®^, Lincoln, NE, USA). The column was filled with a normal phase silica gel (4 g silica RediSep^®^ Rf columns from Teledyne isco^®^, Lincoln, NE, USA). For the first flash chromatography, the column was eluted by a binary gradient mobile phase composed of dichloromethane: methanol (from 0% to 40% of methanol) at a flow rate of 15 mL/min and resulted in the isolation of seven fractions that were used in cell proliferation bioassays (see below). Two fractions (F2 and F3) were selected for a second flash chromatographic fractionation using the same column but with a different mobile phase composed of dichloromethane: methanol (from 10% to 30% methanol) at a flow rate of 10 mL/min. The 3 sub-fractions collected from F2 and the 5 sub-fractions collected from F3 were tested in the cell proliferation bioassay. For proliferation bioassay, LG and NI extracts as well as the chromatographic fractions were dissolved at a concentration of 1 mg/mL in DMSO and adjusted to 10% DMSO (*v*/*v*) with culture medium before to be subjected to proliferation inhibition assay at appropriate concentrations of the tested samples.

### 3.5. ^1^H-NMR-Based Metabolomic Analysis of Tobacco Extracts

All spectra were recorded on a Brüker AV-300 NMR spectrometer operating at a proton NMR frequency of 300.13 MHz. For each sample, 128 scans were recorded following the conditions described by Choi [30]. The resulting spectra were automatically phased, baseline-corrected and manually calibrated at 0.0 ppm using residual solvent signals in MestReNova v8.0.2 (Mestrelab Research, Santiago de Compostela University, Santiago de Compostela, Spain).

### 3.6. Data Analysis

The ^1^H-NMR spectra were aligned and automatically reduced to ASCII files using MestReNova. The data were reduced to integrated regions of equal width (0.04 ppm) over the region δ 0.0 to 12.0 ppm and normalized to the total spectral area in order to suppress trivial separation based on variations in amount of sample. The regions δ 7.26 and δ 4.1–5.3 ppm that correspond to solvents residual signals were excluded from all spectra used for analysis of chloroformic and aqueous extracts, respectively, in order to not compromise the analysis. Principal component analysis (PCA) and orthogonal projections to latent structures discriminant analysis (OPLS-DA) were performed with the SIMCA-P+ software, version 12.0.1 (Umetrics, Umeå/Malmö, Sweden). The degree of separation for each group of data was determined from the scores plot, and the signals that contribute to the composition were obtained from the factor loadings plot. A covariance matrix, generated by the mean-centered method without scaling (subtraction of the column mean from each data point), was used for analysis. OPLS-DA was carried out between *R. fascians*-infected and non-infected tobacco plants to identify metabolites affected by infection. To facilitate interpretation of OPLS-DA a predictive component was calculated after removing the variation explained by orthogonal components and, correlation coefficient [*p*(corr)] were computed for each bin and represented by S-plot. Metabolites (bins) showing *p*(corr) > 0.5 were then considered significant. For all plots, a hostelling 95% confidence ellipse was drawn. Metabolites indicated in Table 3 and Figure 7b were assigned on the basis of chemical shifts and identified from spectral databases and bibliographic search for the chemical shifts of pure compounds putatively present in the ^1^H-NMR spectra of the extracts [21,30].

### 3.7. Gas Chromatography and Mass Spectrometry GC-MS Analysis

GC-MS analyses were performed on a Thermo ITQ 900 system (Thermo Fisher Scientific Inc., Hudson, NH, USA) equipped with an Agilent DB5 capillary column of 30 m × 0.25 mm with a phase thickness of 0.25 μm (Agilent Technologies, Santa Clara, CA, USA). The injector temperature was set to 250 °C, the transfer line to 250 °C and the source to 150 °C; the temperature program was 40 °C for 1 min, 9 °C/min increase rate up to 130 °C, followed by a 2 °C/min increase rate to 230 °C as described by Hamm [23]. The carrier gas was helium, with a column head pressure of 10 psi. The chloroformic and commercially available cembrene (Accros Organics, Geel, Belgium) samples (50 μg/mL) were injected in the splitless mode (2 μL injected, 1 min). The mass spectrometer was operated in electron-impact mode (EI) at 70 eV, in the scan range *m*/*z* 29–400.

### 3.8. Antiproliferative Activity by MTT Assay

The colorimetric MTT viability assay (3-(4,5-dimethylthiazol-2-yl)-2,5 diphenyltetrazolium bromide; Sigma, Diegem, Belgium) was used to determine the overall growth level of each cell line at 72 h as described previously [41] (*n* = 3 with 6 replicates for each concentration).

## 4. Conclusions

In 1997, Vereecke *et al.* have shown that the chemical composition of ethanolic and aqueous extracts from tobacco LG was drastically changed compared to NI tissue. Chlorogenic acid was abundant both in LG and NI tissue, but caffeic acid and another cinnamoyl analog were new in LG. The most pronounced product induced in LG was identified as 7-methyl esculin (7-*O*-methyl-6-*O*-β-d-glucopyranosyl coumarin). It was the first report of the occurrence of this coumarin derivative in tobacco. Since 7-Methyl esculin barely affected the growth of *R. fascians* and was not catabolized, the authors postulate that 7-methyl esculin might locally influence plant cell division, providing appropriate and optimal environmental and nutritional conditions to establish the ecological niche of the invading bacteria. More recently, we have shown that tobacco LG extracts have antioxidant and anti-inflammatory activities [19], as compared to NI plant extracts. All together these study support the occurrence of dramatic metabolic changes in *R. fascians* infected tissues that remain largely unexplored and therefore incite us to better explore LG as a resource for new compounds with promising potential for drug development.

In this study and in order to get a global view of these metabolic changes, non-targeted metabolomic analysis of aqueous and chloroform extracts of LG and NI tobacco was carried out by ^1^H-NMR coupled to PCA and OPLS-DA. Aqueous extract profiling exhibits modifications mainly in the primary metabolites and in some polyphenolics. Certainly, the synthesis of primary metabolites seems to be a quite general mechanism observed during plant-pathogen interactions and the tobacco cell response is probably not specific to *R. fascians* infection suggesting a role of these compounds in the established the ecological niche which is the standing goal of the invading bacteria. Unexpectedly, main modifications occurring in LG chloroformic extract concern secondary metabolites, and GC-MS evidenced alterations in the diterpenoids family, including the cembrenoid family that is known to have antiproliferative activity against cancer cells. A bio-guided fractionation of LG chloroformic crude extract yield to semi-purified fractions, which show antiproliferative activity in glioblastoma U373 cells with an IC_50_ value between 14.0 and 2.4 μg/mL. Experiments are now in progress to isolate the active compounds, elucidate it’s (their) structure(s) and investigate the mechanism of action of such compounds as an antiproliferative compounds against human cancer cells.

## Figures and Tables

**Table 1:** The one-way ANOVA analysis of *Rhodococcus. fascians* infected and non-infected tobacco extracts PCA scores based on ^1^H-NMR signals changes.

Extracts	DF	F	*p*-value
Polar metabolites	8	0.15	0.71
Non-polar metabolites	12	636.3	4.37 × 10^−11^

DF: describes the degrees of freedom and F the fisher coefficient.

**Table 2:** The summary of PCA and OPLS-DA models.

Multivariate data analysis models	A	*n*	*R*^2^	*Q*^2^
**PCA**				
**Non-polar metabolites**	3	13	0.99	0.99
**Polar metabolites**	6	9	0.99	0.65
**OPLS-DA**				
**Polar metabolites**	1+4	9	0.98	0.93

A describes the model components; *n*, the number of observations; *R*^2^, the total variation explained; *Q*^2^, the predictability and statistical validity of model (*Q*^2^ > 0.5 is considered significant).

**Table 3:** The VIP (variable importance in the projections) values of the major metabolites change in *Rhodococcus fascians*-infected tobacco (LG) contributing for the separation in the OPLS-DA score plots.

Significant polar metabolites	^1^H chemical shifts (ppm)	VIP values
5,*O*-caffeoylquinic acid	3.73 (dd, *J* = 9.7 Hz, 3.5 Hz, H-4)	6.48
Inositol	3.61 (t, *J* = 9.7 Hz, H-4 and 6)	4.94
Proline	2.34 (m, H-3)	4.61
Malic acid	2.80 (dd, *J* = 15.9 Hz, 4.5 Hz, H-*b*)	2.33

**Table 4:** Determination of *in vitro* growth activity of LG and NI tobacco crude extracts.

Cell lines	Infected plants (IC_50_ μg/mL)				Non infected plants (IC_50_ μg/mL)			
	
	*Water*	*Methanol*	*Chloroform*	*Hexan*	*Water*	*Methanol*	*Chloroform*	*Hexan*
A549	NA	>100	69.8 ± 0.9	>100	NA	NA	>100	>100
MCF-7	NA	NA	85.6 ± 1.0	>100	NA	NA	>100	NA
PC3	NA	NA	84.5 ± 4.3	>100	NA	NA	>100	NA
U373	NA	>100	58.9 ± 2.0	>100	NA	NA	>100	>100

NA indicates non active fraction.
